# Automated total and vessel-specific coronary artery calcium (CAC) quantification on chest CT: direct comparison with CAC scoring on non-contrast cardiac CT

**DOI:** 10.1186/s12880-022-00907-1

**Published:** 2022-10-14

**Authors:** Jie Yu, Lijuan Qian, Wengang Sun, Zhuang Nie, DanDan Zheng, Ping Han, Heshui Shi, Chuansheng Zheng, Fan Yang

**Affiliations:** 1 Department of Radiology, Tongji Medical College, Union Hospital, Huazhong University of Science and Technology, 1277 Jiefang Ave., Wuhan, 430022 Hubei Province China; 2 Hubei Province Key Laboratory of Molecular Imaging, Wuhan, 430022 Hubei Province China; 3 ShuKun (BeiJing) Technology Co. Ltd., Jinhui Bd, Qiyang Rd, Beijing, 100000 China

**Keywords:** Coronary artery calcium, Coronary artery disease, Artificial intelligence, Tomography, X-ray computed, Thorax

## Abstract

**Background:**

This study aimed to evaluate the artificial intelligence (AI)-based coronary artery calcium (CAC) quantification and regional distribution of CAC on non-gated chest CT, using standard electrocardiograph (ECG)-gated CAC scoring as the reference.

**Methods:**

In this retrospective study, a total of 405 patients underwent non-gated chest CT and standard ECG-gated cardiac CT. An AI-based algorithm was used for automated CAC scoring on chest CT, and Agatston score on cardiac CT was manually quantified. Bland-Altman plots were used to evaluate the agreement of absolute Agatston score between the two scans at the patient and vessel levels. Linearly weighted kappa (κ) was calculated to assess the reliability of AI-based CAC risk categorization and the number of involved vessels on chest CT.

**Results:**

The AI-based algorithm showed moderate reliability for the number of involved vessels in comparison to measures on cardiac CT (κ = 0.75, 95% CI 0.70–0.79, *P* < 0.001) and an assignment agreement of 76%. Considerable coronary arteries with CAC were not identified with a per-vessel false-negative rate of 59.3%, 17.8%, 34.9%, and 34.7% for LM, LAD, CX, and RCA on chest CT. The leading causes for false negatives of LM were motion artifact (56.3%, 18/32) and segmentation error (43.8%, 14/32). The motion artifact was almost the only cause for false negatives of LAD (96.6%, 28/29), CX (96.7%, 29/30), and RCA (100%, 34/34). Absolute Agatston scores on chest CT were underestimated either for the patient and individual vessels except for LAD (median difference: − 12.5, − 11.3, − 5.6, − 18.6 for total, LM, CX, and RCA, all *P* < 0.01; − 2.5 for LAD, *P* = 0.18). AI-based total Agatston score yielded good reliability for risk categorization (weighted κ 0.86, *P* < 0.001) and an assignment agreement of 86.7% on chest CT, with a per-patient false-negative rate of 15.2% (28/184) and false-positive rate of 0.5% (1/221) respectively.

**Conclusions:**

AI-based per-patient CAC quantification on non-gated chest CT achieved a good agreement with dedicated ECG-gated CAC scoring overall and highly reliable CVD risk categorization, despite a slight but significant underestimation. However, it is challenging to evaluate the regional distribution of CAC without ECG-synchronization.

**Supplementary Information:**

The online version contains supplementary material available at 10.1186/s12880-022-00907-1.

## Background

Coronary artery calcium (CAC) score is a robust imaging surrogate of atherosclerosis burden and has been proven to be a useful tool for cardiovascular disease (CVD) risk stratification and primary prevention with well-established clinical evidence [1–3].

CAC is traditionally evaluated on dedicated electrocardiography (ECG)-gated non-contrast cardiac CT and reported as a total Agatston score at the patient level [4]. However, several studies have indicated that the number of coronary arteries with positive CAC added to the total Agatston score for risk stratification and prediction of cardiac events [5–9]. Both total Agatston score and regional distribution of CAC should be reported as recommended in the latest CAC data and reporting system (CAC-DRS) [4].

Recently, CAC quantification on non-gated chest CT has emerged as an alternative to standard CAC scoring on ECG-gated cardiac CT, regardless of suboptimal image quality [10–12]. Since cardiovascular risk factors also contribute to non-cardiovascular diseases such as lung cancer, participants would benefit from CAC scoring for assessment of the risk of CVD and lung cancer on chest CT without additional radiation exposure [10–13]. Previous studies implied that the calcium regional distribution combing with total Agatston score should be evaluated across non-gated and ECG-gated scans, however, it has not been validated in direct comparison with measurements on ECG-gated CT scans [9, 14].

It is time-consuming and challenging for manual CAC scoring and even more difficult to identify the involvement of individual coronary arteries on non-gated chest CT since there are frequent motion artifacts and noise. Algorithms based on artificial intelligence (AI) have shown the potential for efficient CAC scoring on chest CT [11, 15–18]. A few latest studies have validated the accuracy of AI-based vessel-specific or even lesion-specific CAC quantification on ECG-gated cardiac CT [4, 19, 20]. However, it remains unknown whether AI-based automated vessel-specific CAC assessment on chest CT is comparable to dedicated measurements on cardiac CT and how the CAC distribution would affect the accuracy of quantification of total CAC score. The comprehensive comparison is essential because it would be helpful for the improvement of the AI algorithm and facilitate the utility of automated CAC scoring on routine chest CT examinations.

Therefore, this study aimed to investigate the performance of AI-based automated quantification of the total CAC score and risk categorization and vessel-specific CAC assessment on non-gated chest CT, using standard CAC scoring on ECG-gated cardiac CT as a reference standard.

## Materials and methods

### Study participants

From 1st August 2019 to 31st December 2020, 3982 patients with known or suspected coronary artery disease underwent standard ECG-gated cardiac CT, and 516 of them underwent routine non-contrast non-gated chest CT for lung cancer screening or other diagnosis purposes, such as routine physical examination, in the same session. The median time gap between ECG-gated cardiac CT and non-gated chest CT was 2 days (interquartile range [IQR] 1–4 days) and ranging from 0 to 18 days.

**Fig. 1:**
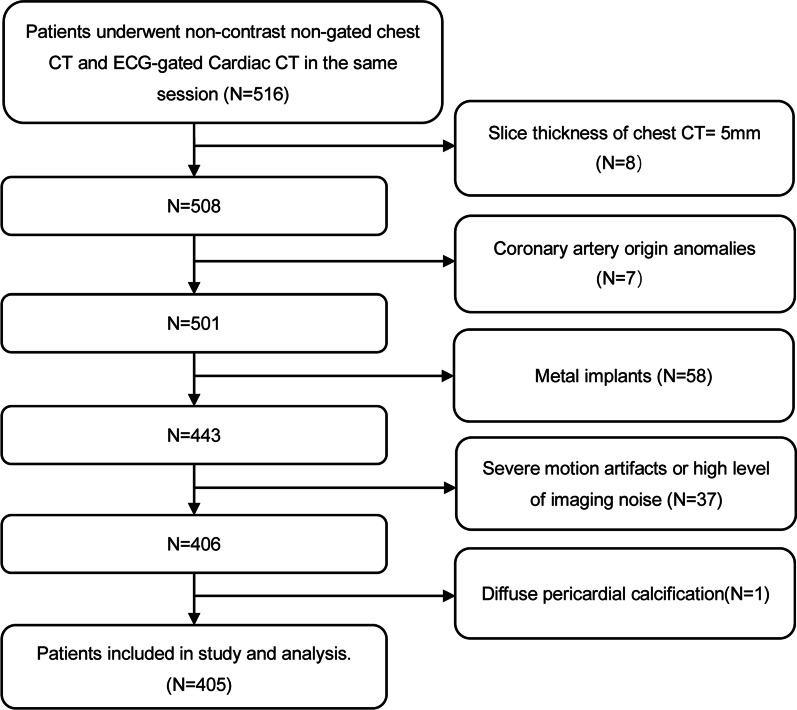
Flow chart showing inclusion and exclusion of patients

The exclusion criteria are shown in Fig. 1. Eight patients were excluded because the thin slices images are not available, and seven patients were excluded because of coronary origin anomalies. Metal implantation (n = 58) included coronary stent (n = 45), coronary bypass grafts (n = 6), prosthetic valves (n = 5) and permanent pacemakers (n = 4). Since motion artifacts are an inherent limitation of chest CT without ECG synchronization, patients with motion artifacts were not intentionally excluded, only those with extremely severe motion artifacts and a high level of noise that it was difficult to identify coronary artery and CAC even manually (n = 37). Finally, 405 patients were included in this study (Fig. 1).

ECG-gated cardiac CT and diagnostic chest CT were retrospectively and anonymously collected. The need for informed consent was waived by our Institutional Review Board.

### CT imaging protocols

#### ECG-gated non-contrast cardiac CT

The acquisition protocols of dedicated prospective ECG-gated non-contrast cardiac CT are summarized in Table 1. Standard cardiac CT scan was in sequential mode at a tube voltage of 120 kVp and automated tube current modulation (CARE Dose 4D, Siemens Healthineers, Erlangen, Germany) with sets of reference mAs at 80 and a matrix size of 512 pixels. Trans axial images were reconstructed using the medium sharp convolution kernel (Qr36) with a 3.0-mm section thickness and an increment of 1.5 mm. The center phase of the cardiac cycle to trigger the scan was 40% or 70% of the R-R interval according to the patient’s heart rate.

**Table 1:** Protocols of non-gated chest CT and ECG-gated CT

	Chest CT			ECG-gated Cardiac CT
Scanner	SIEMENS Definition	SIEMENS Definition AS	TOSHIBA AquilionOne	SIEMENS Force
N	202	98	105	405
Rot.(s)	0.5	0.5	0.5	0.25
Exposure Time/Rot.(s)	0.5	0.5	0.5	0.15
Pitch	0.95	1.2	0.83	NA
Collimator (mm)	1.2 × 24	0.6 × 64	0.5 × 32	1.2 × 40
Inplane resolution (mm)	0.77 × 0.77	0.73 × 0.73	0.78 × 0.78	0.33 × 0.33
Convolution Kernel	B30f, B35f	B30f, B35f	FC18	Qr36f
DLP(mGy*cm)^a^	186 (164–243)	284 (213–347)	338 (251–416)	35 (28–33)
EDvol(mSv)^b^	2.6 (2.3–3.4)	4.0 (3–4.9)	4.7 (3.5–5.8)	0.5 (0.4–0.5)
Slice thickness (mm)	1.5	1.5, 2.0	2.0	3.0
Increment (mm)	1.2	1.2,1.5	1.5	1.5

^a^median (interquartile range); ^b^median (interquartile range)

*CAC* Coronary artery calcium, *CT* Computed tomography, *ECG* Electrocardiography, *DLP* Dose-length production, *ED* Effective dose

#### Non-gated non-contrast chest CT

As shown in Table 1, non-contrast non-gated chest CT was performed on multiple scanners. The tube voltage was 120 kVp, and the tube current was modulated automatically. Images were reconstructed with a standard soft convolution kernel with slice thickness varying from 1.5 to 2.0 mm and a matrix size of 512 pixels.

#### Radiation dose

To calculate the effective dose (ED) of the cardiac and chest CT, the dose-length product (DLP) was multiplied by the conversion coefficient (k = 0.014) [21, 22].

### CAC measurements

#### CAC scoring on ECG-gated cardiac CT

CAC scores on ECG-gated cardiac CT were evaluated using semiautomatic software on a dedicated workstation (CaScoring, Syngo. Via VB20; Siemens Healthineers, Erlangen, Germany) with a threshold of 130 HU and minimum area of 1.0 mm^2^ [23]. CAC lesions were manually annotated to the left main trunk (LM), left anterior descending artery (LAD), circumflex (CX), and right coronary artery (RCA) by two radiologists (J.Y and H.SH) with over 10 years of experience in cardiovascular imaging independently. The discrepancies were resolved through discussion and CAC scores were then averaged. The number, volume (mm^3^), effective calcium mass (mg), and Agatston score of CAC were recorded for the patient and individual arteries. The Agatston score of each coronary artery and total Agatston score were used for analyses and served as a reference.

#### AI-based CAC scoring on non-gated chest CT

All non-gated chest CT images were imported into commercially available AI-based automated CAC scoring software (CACScoreDoc; ShuKun Technology, Beijing, China), which was implemented at a regular workstation to calculate the CAC score. Details of the AI-based algorithm are described in Additional file 1. Briefly, this software was based on a deep learning algorithm and was trained on multi-scanner and multi-hospital anonymized external chest CT databases. A two-step deep learning workflow was used to accomplish cardiac segmentation, and coronary artery calcified lesion segmentation and classify them according to the branches [see Additional file 1: Fig. 1]. Based on the segmentation results, the volume, mass, and Agatston scores within the coronary tree (LM, LAD, CX, and RCA) were calculated and recorded.

#### Manual CAC scoring on chest CT

The distribution of CAC involvement in each coronary artery was also manually evaluated on non-gated chest CT, that is a previous test to assess the feasibility and possible effects without ECG-synchronization. More details are included in the Additional file 2.

### Number of vessels with CAC

Agatston scores were rounded and classified as Agatston score = 0 and Agatston score ≥ 1 to determine the absence and presence of CAC. The number of vessels with CAC was calculated as an ordinal variable indicating the cumulative involvement of the LM, LAD, CX, and RCA on both chest and cardiac CT, and patients were classified into N1(1-vessel), N2(2-vessels), N3(3-vessels), and N4 (4-vessels) according to CAC-DRS, and N0 indicates no calcium involvement of arteries [4, 9].

### CAC risk categories

The participants were classified into four risk groups based on the total Agatston score on non-gated chest CT and ECG-gated cardiac CT according to CAC-DRS: very low risk (A0, CAC = 0), mild risk (A1, CAC = 1–99), moderate risk (A2, CAC = 100–299), and moderate to severe risk (A3, CAC ≥ 300) [4].

### Statistical analysis

Normally distributed continuous data are presented as the mean ± standard deviation and non-normally distributed data as median and IQR (25th–75th percentile).

Cohen’s linearly weighted kappa (κ) was calculated to assess the reliability of AI-based classification of the number of vessels with CAC in comparison to measurement on cardiac CT. The per-vessel sensitivity, specificity, and the false-negative and false-positive rates of automated identification of involved coronary arteries on chest scans were calculated. The mismatched AI-based Agatston scores and risk category shifts on non-gated chest CT were reviewed by two radiologists. Potential causes for errors were identified via discussion.

In patients or individual arteries with positive CAC on ECG-gated cardiac CT, the correlation of the Agatston score between non-gated chest CT and ECG-gated cardiac CT was assessed using the Spearman correlation coefficient (ρ), and the Agatston score difference between the two scans was evaluated using the Wilcoxon signed-rank test.

Agreement and reliability between the two scores were reported following previously published guidelines [24]. Bland-Altman plots with 95% limits of agreement were used for the evaluation of the agreement of the Agatston score between the two methods. Due to the half-normal distribution of CAC difference between the two methods, the 95% limits of agreement in Bland–Altman plots were calculated for total and vessel-specific Agatston scores using a regression model in previous studies [25].

The reliability of AI-determined Agatston risk categorization on chest CT was evaluated using Cohen’s linearly weighted kappa (κ) in comparison to the reference standard on cardiac CT, and the proportion of agreement was determined. The kappa was interpreted following a previous report [26].

Two-tailed *P* values < 0.05 were considered statistically significant. Statistical analyses were conducted using SPSS V27.0 (IBM Corp, Armonk, NY, USA).

## Results

### Patient characteristics

Characteristics of all 405 included patients are shown in Table 2. The mean age was 59.6 ± 11.8 years, and 42.0% were female. The median body mass index (BMI) was 24.8 (IQR 22.8–27.4).

**Table 2:** Patient characteristics

Characteristic	
Age (years)^a^	59.6 ± 11.8^a^
Sex	
Female n (%)	170 (42.0)
Male n (%)	235 (58.0)
Body mass index (kg/m^2^)^b^	24.8 (22.8–27.4)^b^
Hypertension n (%)	130 (32.1)
Systolic blood pressure (mmHg)^a^	127 ± 17^a^
Hyperlipidaemia n (%)	180 (44.4)
Diabetes n (%)	40 (9.9)
Tobacco abuse	
Current smoker n (%)	154 (38.0)
Ex-smoker n (%)	24 (5.9)
Family history of CVD n(%)	24 (5.9)

^a^mean ± standard deviation, ^b^median (interquartile range)

*CAC* Coronary artery calcium, *ECG* Electrocardiography, *CVD* Cardiovascular disease

### Number of vessels with CAC

**Fig. 2:**
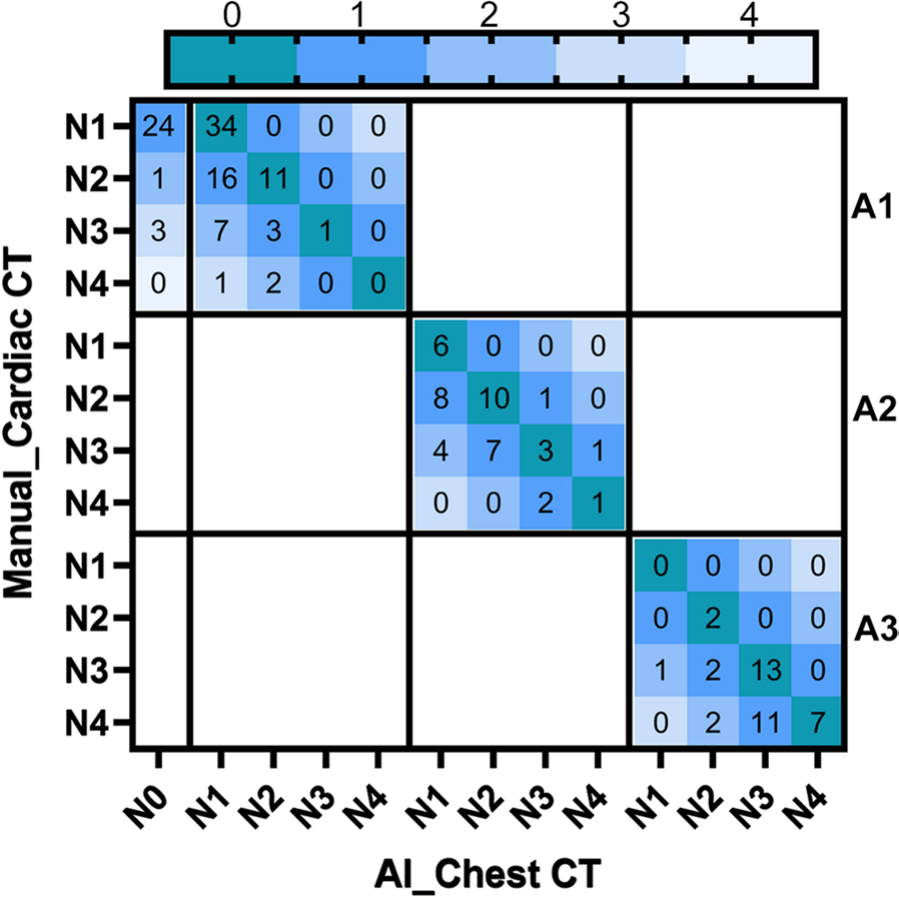
Comparison of the number of vessels with CAC between non-gated chest CT and ECG-gated cardiac CT. The patients with positive CAC are classified into 3 groups according to standard CAC scores on ECG-gated cardiac CT (A1:1–99,A2:100–299,A3: ≥ 300).Note that among patients of category A1, twenty-eight are falsely identified as N0 on chest CT. The color bar on the top indicates the category shifts of the number of involved vessels. (0 = correctly identification, 1 = 1vessel not identified, 2 = 2 vessels not identified, 3 = 3vessels not identified and 4 = 4 vessels are not identified)

Table 3 compares the classification of the number of vessels with CAC between chest CT and ECG-gated cardiac CT. The AI-based algorithm showed moderate reliability for the number of involved vessels in comparison to that on dedicated cardiac CT (κ = 0.75,95%CI 0.70–0.79, *P* < 0.001) and an assignment agreement of 76%. The patients were divided into three groups according to Agatston score on ECG-gated cardiac CT. The overall accuracy of assignment of number of involvement was 44.7%, 46.5%, and 57.9% on non-gated chest CT for patients with ECG-gated CAC score of 1–99 (A1), 100–299 (A2), and ≥ 300 (A3) respectively (Fig. 2).

**Table 3:** Automated number of involved vessels on chest CT versus measurements on cardiac CT

	Refernce Number of vessels_Cardiac CT					
Automated Number of vessels_Chest CT	N0	N1	N2	N3	N4	Total
N0	**220**	24	1	3	0	248
N1	1	**40**	24	12	1	78
N2	0	0	**23**	12	4	39
N3	0	0	1	**17**	13	31
N4	0	0	0	1	**8**	9
Total	221	64	49	45	26	405

*N0* No involved vessels with CAC, *N1* Patients with 1-vessel CAC, *N2* Patients with 2-vessel CAC, *N3* Patients with 3-vessel CAC, *N4* Patients with 4-vessel CAC; Bold values indicate concordant classification between the two methods

Per-vessel analyses showed that the AI-based algorithm yielded a sensitivity of 40.7% (22/54 vessels), 82.2% (134/163 vessels), 65.1% (56/86 vessels) and 65.3% (64/98 vessels), with false-negative rates of 59.3% (32/54 vessels), 17.8% (29/163 vessels), 34.9% (30/86 vessels), and 34.7% (34/98 vessels) for LM, LAD, CX and RCA respectively, and a specificity of 99.2% (348/351 vessels), 98.4% (238/242 vessels), 99.4% (317/319 vessels) and 100% (307/307 vessels) for LM, LAD, CX and RCA respectively. False positives were observed in 0.9% of LM (3/351 vessels), 1.7% of LAD (4/242 vessels), 0.6% of CX (2/319 vessels).

**Fig. 3:**
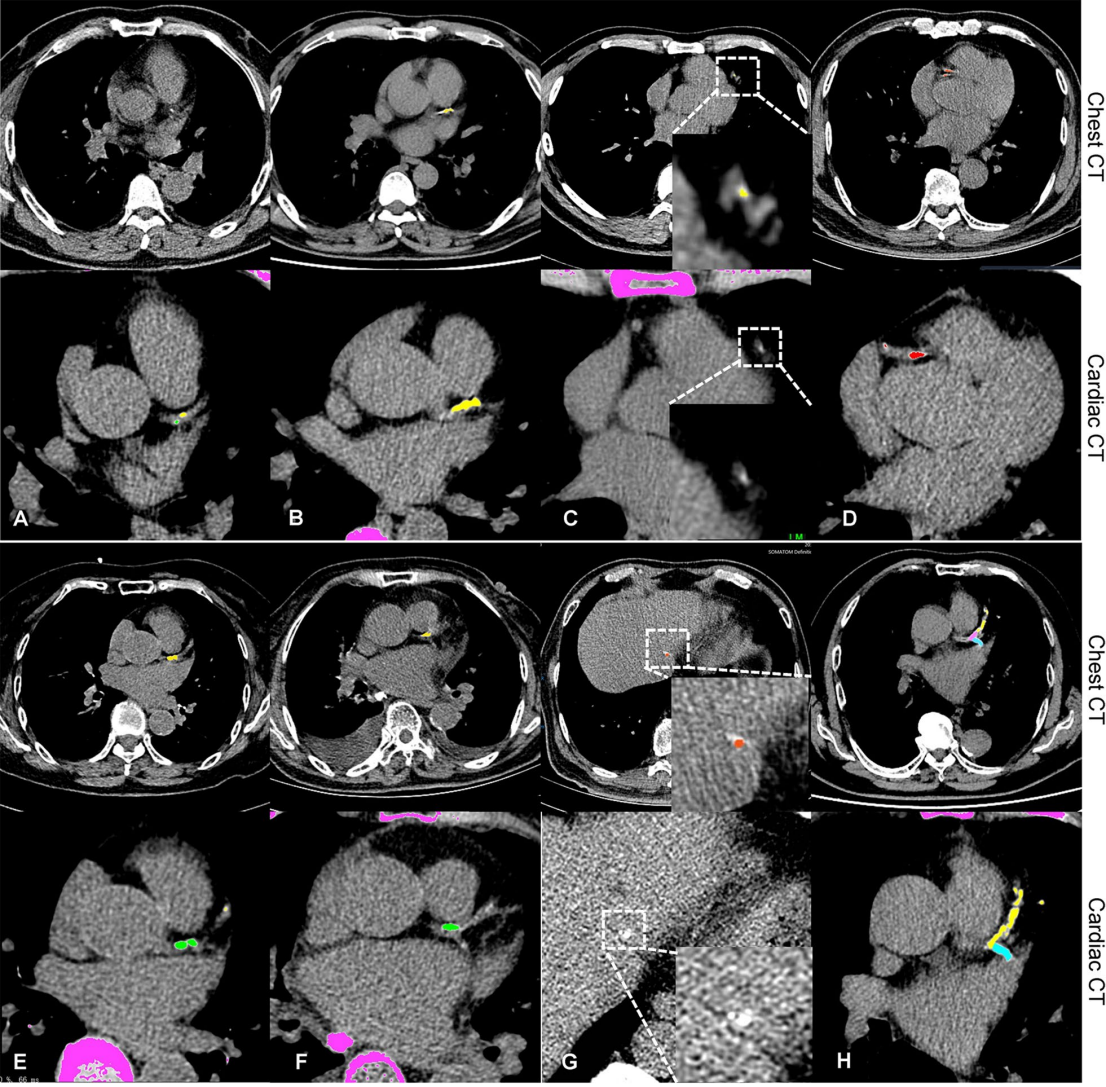
Examples show potential causes of discordances of Agatston scores between chest CT and cardiac CT. For each case(A-H), the upper row is AI-based scoring on chest CT, and the lower row is the manual measurement on cardiac CT. CAC lesions were annotated with different colours (i.e., green for LM, yellow for LAD, blue for CX, red for RCA, and pink for non-coronary calcifications). **A**. False negatives due to motion artifact, **B**. Underestimation due to motion artifact (Agatston score:57.7 versus 225.0 between chest CT and cardiac CT). **C**. False positive due to image noise on chest CT, it is not identified because of tiny size and lower density (< threshold of 130HU) on standard cardiac CT. **D**. Overestimation due to motion artifact (Agatston score:126.0 versus 88.0 between chest CT and cardiac CT). **E**. Segmentation error, CAC of LM (green on cardiac CT) is falsely identified as calcification of LAD (yellow on chest CT). **F**. Segmentation error combined with motion artifact, CAC of LM (green on cardiac CT) is wrongly identified as CAC of LAD (yellow on chest CT) and blurred due to motion artifact. **G**. Segmentation error, calcification of liver is misidentified as CAC of RCA. **H**. Segmentation error, part of CAC of LAD is misidentified as non-coronary calcification, resulting in underestimation (Agatston score:48.1 versus 713.2 between chest CT and cardiac CT)

For false negatives of LM CAC, the main causes were motion artifacts (56.3%, 18/32 vessels), and segmentation error (43.8%, 14/32 vessels). The motion artifact was the only cause for false negatives of LAD, CX, and RCA. The leading cause of false positives was segmentation error, that is, misidentification of calcium on adjacent coronary arteries or structures such as the aorta, pericardium, rib, or lymph node. None of the RCA with no CAC on cardiac CT was falsely identified as calcified artery on chest CT. Figure 3 shows some examples of discordant pairs between chest CT and ECG-gated cardiac CT.

### Quantification of absolute CAC score

In patients with positive CAC on ECG-gated cardiac CT(n = 184), the correlation between chest CT and ECG-gated cardiac CT for the total Agatston score was very strong (Spearman coefficient ρ = 0.95, *P* < 0.001). Among coronary arteries with positive CAC, the correlation between the two methods was strong for the Agatston score of LAD, CX, and RCA (ρ = 0.88, 0.80, and 0.81 respectively, all *P* < 0.001), but only moderate for the Agatston score of LM (ρ = 0.58, *P* < 0.01).

The absolute Agatston scores at the patient and vessel levels are summarized in Table 4. Analyses for each risk category showed that the difference between the two scores tended to increase as CAC increased. Total Agatston score was significantly underestimated for all participants and each CAC risk group, especially in the category of ≥ 300 (median 570.8 vs. 519.1, *P* < 0.01), as well as the CAC score on RCA (median 188.5 vs. 78.8, *P* < 0.01).

**Table 4:** Automated total and per-vessel non-gated Agatston scores versus ECG-gated measurements

		Agatston_ECG	Agatston_AI	*P* value
All CAC > 0^a^	Total (n = 184)	73.1(26.2–256.2)	63.3(11.6–221.8)	< 0.001
	LM (n = 54)	41.2(9.3–114.7)	0(0–50.4)	< 0.001
	LAD (n = 163)	56.6(15.3–147.2)	45.7(8.5–142.8)	0.18
	CX (n = 86)	31(5.7–81.9)	17.5(0–73.9)	< 0.01
	RCA (n = 98)	46(10–141.1)	14.8(0–79.5)	< 0.001
Risk Category^b^				
1–99	Total (n = 103)	30.6(9.9–51.9)	13.9(0–39.5)	< 0.001
	LM (n = 16)	8.3(1.9–27.3)	0(0–0)	< 0.001
	LAD (n = 83)	17.1(4.4–38.8)	12(0–32.4)	< 0.01
	CX (n = 32)	9.3(2.7–32.1)	0(0–21)	< 0.001
	RCA (n = 37)	9.9(2.2–27.2)	0(0–14.4)	< 0.001
100–299	Total (n = 43)	163.3(140–245.9)	142.4(95–223.1)	0.02
	LM (n = 15)	41.4(15.3–82.1)	0(0–49.2)	0.11
	LAD (n = 42)	126.9(77.5–151.2)	99.7(43.8–145.5)	0.20
	CX (n = 19)	23.7(12–33.9)	5.3(0–55.3)	0.26
	RCA (n = 25)	50.7(11.7–105.2)	14.3(0–93.3)	0.02
≥ 300	Total (n = 38)	570.8(423.1–889.2)	519.1(310–922.7)	< 0.01
	LM (n = 23)	103.2(58.1–220.6)	9.4(0–122.8)	0.08
	LAD (n = 38)	270.5(141.8–444.7)	298(150.6–457.1)	0.24
	CX (n = 35)	114.4(32.2–199.1)	81.5(17.4–184.7)	0.24
	RCA (n = 36)	188.5(104.2–318.2)	78.8(18–222)	< 0.001

*ECG* Electrocardiography, *AI* Artificial intelligence, *CAC* Coronary artery calcium

**Fig. 4:**
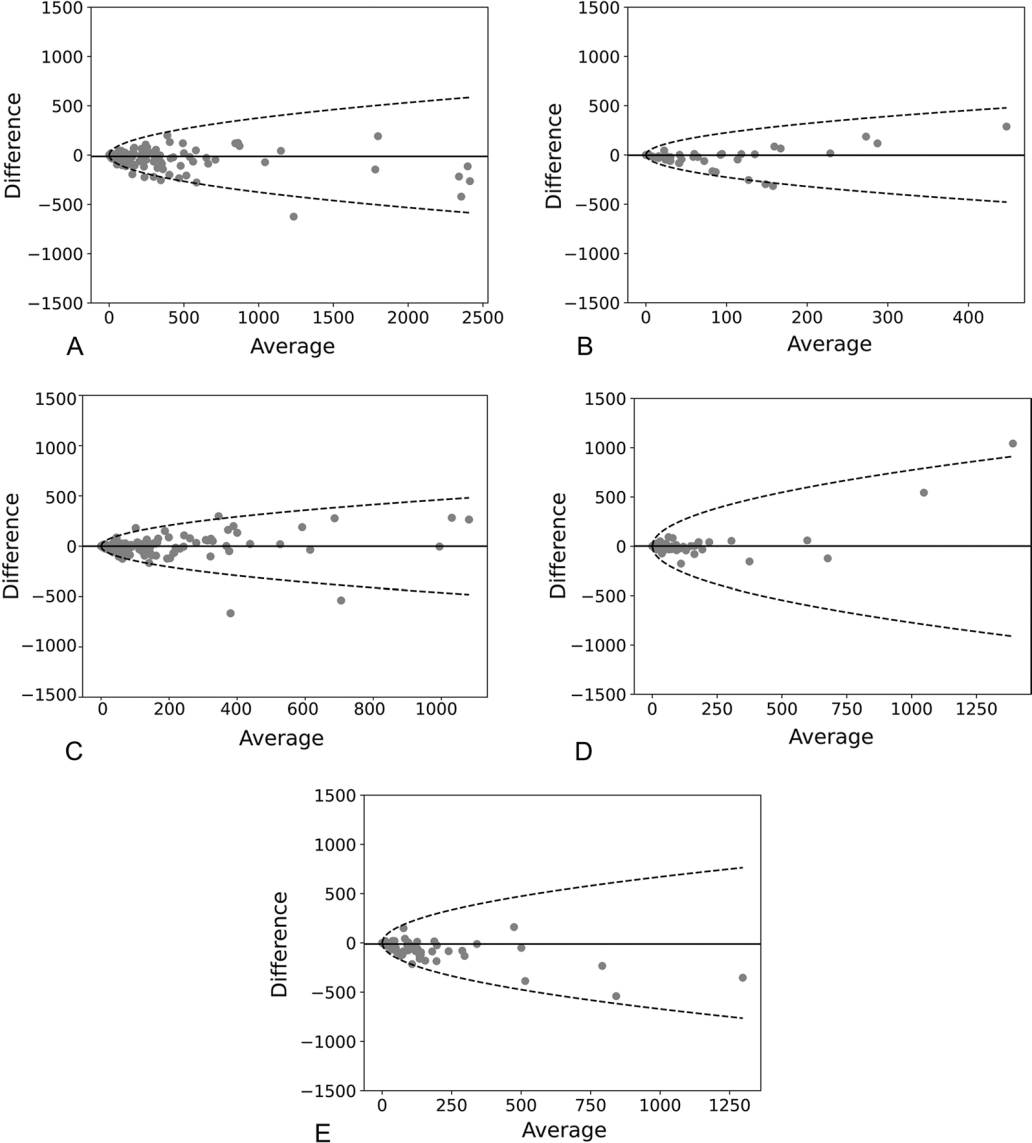
Bland–Altman plots of Agatston scores of CAC. Dashed lines show 95% limits of agreement. The average score from ECG-gated CAC scoring and AI-based automated quantification on non-gated CT is plotted against the difference between the two methods, the difference is Agatston score on chest CT minus the measurement on cardiac CT. The plots reveal underestimated calcium scores with the automated method on non-gated CT and an increasing difference with a high average score. A regression of absolute difference (X) is performed, and 95% limits of agreement (Y) are calculated as follows: Y = (− 8.25 + 5.26 *X^0.5^)*1.96 *(π/2)^0.5^ for Total Agatston score (**A**), Y = (− 17.53 + 10.96*X^0.5^)*1.96 *(π/2)^0.5^ for Agatston scores of Left main trunk (LM) (**B**), Y = (− 19.95 + 7.38*X^0.5^)*1.96 *(π/2)^0.5^ for Agatston scores of left anterior descending artery (LAD) (**C**), Y = (− 50.70 + 13.76*X^0.5^)*1.96 *(π/2)^0.5^ for Agatston scores of circumflex (CX) (**D**) and Y = (− 19.22 + 10.07*X^0.5^)*1.96 *(π/2)^0.5^ for Agatston scores of Right coronary artery (RCA)(E) respectively

Bland–Altman plots showed a slightly systematic underestimation of the total Agatston score but relatively narrow limits of agreement (Fig. 4A). For vessel-specific CAC scores, Bland–Altman plots showed good agreement between non-gated chest CT and standard ECG-gated cardiac CT for CAC scores on LAD with narrower limits of agreement (Fig. 4C). In contrast, there was a slight but significant underestimation of AI-based CAC scores of LM, CX, and RCA compared to standard measurements on ECG-gated cardiac CT. Most discordant pairs were below the median bias, and the limits of agreement were relatively wider (Fig. 4B, D, E).

### CVD risk categorization

The confusion matrix in Table 5 compares the CVD risk categorization based on the Agatston score between the two methods. AI software assigned 351 patients to correct Agatston risk stratification defined by the ECG-gated Agatston score with a linearly weighted κ value of 0.86 (95%CI 0.83–0.90; *P* < 0.001), and an assignment agreement of 86.7%.

**Table 5:** Automated Agatston risk categorization on chest CT comparing the standard reference on cardiac CT

	Reference Agatston score_cardiac CT						
Automated Agatston score _chest CT	0	1–99	100–299	≥ 300	Total	Shift downward	Shift upward
0	**220**	28	0	0	248	28	0
1–99	1	**73**	12	0	86	12	1
100–299	0	2	**27**	7	36	7	2
≥ 300	0	0	4	**31**	35	0	4
Total	221	103	43	38	405	47	7

Bold values highlight the concordant classification between the two methods

In all fifty-four misclassified patients, forty-seven shifted downward and seven shifted upward by one category. Regarding the presence and absence of CAC, the per-patient false-negative rate was 15.2% (28/184) and the per-patient false-positive rate was 0.5% (1/221).

## Discussion

In this study, AI-based automated Agatston scores on non-gated chest CT were compared with standard measurements on ECG-gated cardiac CT at the patient and vessel levels. The AI-based method only yielded moderate reliability for the identification of the number of arteries with CAC on chest CT, with a high false-negative rate of LM, CX, and RCA with CAC except for LAD. Nevertheless, the agreement of automated quantification of the total Agatston score on chest CT was good in comparison to standard ECG-gated CAC scoring, and the reliability for risk categorization was high.

Although AI-based algorithms have yielded an almost perfect vessel-specific CAC assessment on EC-gated cardiac CT [19, 20], the performance could be substantially reduced without EG-synchronization. LM showed a high false negative rate and lower Agatston score correlation to the reference standard compared to other vessels. It is difficult for automated distinguishing CAC on LM from lesions on adjacent LAD and CX calcium or aortic, mitral valvular lesions on chest CT, and even for manual CAC detection on ECG-gated cardiac CT, [10, 13, 14, 17, 18] or manual measurements on chest CT [see Additional file 2: Table 1]. Among all coronary arteries, RCA was more susceptible to motion artifacts than other arteries due to its higher velocity during the cardiac cycle, which is sometimes unevaluable even with ECG triggering [27]. The false negatives of CAC involvement in individual coronary arteries would result in the underestimation of the number of involved vessels, especially for the N3(3-vessels) and N4(4-vessels) groups. Given that CAC in LM and increased number of involved vessels may add an incremental prognostic value to the total Agatston CAC score, this would be a specific shortcoming of chest CT for per-vessel CAC scoring compared to dedicated cardiac CT [11, 14, 19, 20]. A motion-correction algorithm is desirable and segmentation accuracy should be improved for the AI algorithm, as indicated in a previous phantom study [28].

Further analyses found that the underestimation of the number of involved vessels most frequently occurred in patients with Agatston score ranging from 1 to 300, especially in patients with the CAC category of 1–99. Without ECG-triggering, the coronary artery involvement with smaller or less dense calcium would be more easily misclassified due to motion artifact, imaging noise, or partial volume effects. The accuracy of the number of involved vessels was similar even with manual measurement within intermediate CAC scores [see Additional file 2: Table 2 and Fig. 1). Several previous studies indicated that the regional calcium distribution provided significantly incremental prognostic value in patients with intermediate Agatston scores (1–300), but not in Agatston scores of > 300 [8, 9, 29]. Therefore, this would be a major drawback of non-gated vessel-specific CAC scoring for risk stratification and prognosis prediction.

Although there was only moderate reliability in the assessment of the regional distribution of CAC, automated quantification of Agatston score at the patient level on chest CT showed a good agreement in comparison to cardiac CT. This may be attributed to considerable heterogeneity between the number of involved vessels and total Agatston score [5, 7, 9]. The majority of patients with a Agatston category of 1–99 have CAC in one vessel, and 4-vessel CAC mainly contributed to a Agatston score of > 300 [9, 29]. Among all four main coronary arteries, LAD was the most common location of positive CAC. Automated quantification of CAC was more correct in LAD than measurements in other involved arteries. Moreover, the effects of underestimation of individual CAC scores will be offset by summing all per-vessel CAC scores together. Taking together, regardless of the substantial underestimation of vessel-specific CAC, total Agatston score on chest CT was accurate using the AI-based algorithm.

It should be emphasized that the total Agatston score rather than the number of involved vessels is a major metric of CAC and an established surrogate for CVD risk stratification, and there is no sufficient evidence to change risk stratification based on the number of involved coronary arteries [4]. From this point of view, it is of clinical significance that automated quantification of CAC score on chest CT was comparable to standard ECG-gated CAC scoring, despite a slight but systemic underestimation (median difference − 12.5). The findings were similar to the results in previous reports on chest CT with a high-temporal high-pitch CT, but the difference between the two scores was relatively larger in the current study [30, 31]. Nevertheless, it is of note that the imaging protocols of chest CT in our study were much more various. The results of the current CT were in line with the previous study with that of multiple protocols and strengthened the previous conclusion by direct comparison to dedicated ECG-gated cardiac CT [17]. Since image acquisition protocols may not necessarily satisfy the 2016 SCCT guidelines, it is meaningful that the AI-based per-patient CAC scoring on daily routine chest CT examination across multiple protocols was comparable to the measurements on dedicated ECG-gated CT [32].

Compared to the reference on ECG-gated cardiac CT, the reliability of non-gated chest CT for CVD risk categorization was strong based on the total Agatston score (agreement of assignment 86.7% and weighted κ 0.86). This result was consistent with previous studies that compared manual CAC scoring on chest CT in comparison to ECG-gated cardiac CT or manual measurement on non-gated CT [15–17, 33]. According to a standard reference on cardiac CT, AI misclassified 28 patients into the category of zero with a per-patient false-negative rate of 15.2%. Since a CAC score of zero has a special power for the prediction of very low CVD risk and the CAC-based CVD category was recommended for statin treatment, the underestimation of the CVD risk category would impact the initial therapy decision or preventive strategy [4, 34, 35]. Nevertheless, this may be a common shortcoming of AI-based algorithms for CAC scoring [15, 17]. In this study, most patients (129/183) with discordant total Agatston scores between the two methods were assigned to correct risk categories although there was a downward shift trend of risk category. These results further indicated that the error of the absolute total Agatston score may be acceptable for risk categorization, only those near the cutoff would lead to the shift of category and may have potential effects on clinical practice [30, 31, 36].

There were several limitations to this study. First of all, it is a retrospective study with a small sample size, nevertheless, the distribution of the number of vessels with CAC and combing Agatston category was similar to previous reports [9]. Although it is difficult to identify the number of involved vessels in non-gated CT even with manual visualization, it is essential to investigate longitudinal prognosis value of the automated Agatston score and the regional distribution of CAC on non-gated chest CT. Second, the thin slice thickness images were directly used for CAC scoring, this would be the concern of bias, nevertheless it might be reasonable since previous study showed the accuracy of thin and standard 3-mm slices are similar [37]. Last, Standard rather than low-dose non-gated chest CT was performed in the study, although the radiation dose was close to the recommendation in guidelines for lung screening CT [38, 39].

## Conclusion

AI-based automated quantification of CAC scores from non-gated chest CT was comparable to the standard CAC scoring using ECG-gated CT with good agreement and high reliability. However, it is challenging to evaluate vessel-specific CAC without ECG synchronization. It would support CAC quantification on non-gated CT as a clinical approach for cardiovascular risk prediction integrated with chest CT, but the motion-correction algorithm and more powerful segmentation are desirable and eligible participants should be selected.

## Supplementary Information

**Additional file 1:** Development of AI-based automatic CAC on chest CT.

**Additional file 2:** Manual measurement of Coronary artery calcium (CAC) distribution on chest CT.

## Data Availability

The datasets used and/or analyzed during the current study are available from the corresponding author on reasonable request.

## Abbreviations

CAC: Coronary artery calcium.
CVD: Cardiovascular disease
ECG: Electrocardiography
CAC-DRS: Coronary artery calcium data and reporting system
AI: Artificial intelligence
IQR: Interquartile range
ED: Effective dose
DLP: Dose-length product
LM: Left main trunk
LAD: Left anterior descending artery
CX: Circumflex
RCA: Right coronary artery
BMI: Body mass index
CI: Confidence interval
