# Supplementary material for: Automated total and vessel-specific coronary artery calcium (CAC) quantification on chest CT: direct comparison with CAC scoring on non-contrast cardiac CT
Source: BMC Med Imaging. 2022 Oct 14;22:177. doi: 10.1186/s12880-022-00907-1 (PMC9563469; doi:10.1186/s12880-022-00907-1)
Supplement: Supplementary file 1 — Additional file 1: Development of AI-based automatic CAC on chest CT. [file 12880_2022_907_MOESM1_ESM.docx]

**Additional File 1: Development of AI-based software for CAC scoring on chest CT**

To develop the AI-based automatic calcium scoring method, a dataset consisting of 1962 non-contrast chest CT scans was retrospectively collected. Among which, 51(2.6%) scans were excluded due to poor image quality or severe artifact. All the CT images were in DICOM format, and the scans were acquired from GE, Siemens, Philips, and Toshiba equipment (n=643, 714, 540,14 respectively) using 16-, 64-, 128-, 256- or 320-detector row CT scanners. The slice thickness ranged from 0.625mm to 5mm. Tube voltage was set to 120kVp.

In an image annotation center, to establish a reference standard, the cardiac area and calcified lesions were labeled with semi-automated software (ITKSNAP, <http://www.itksnap.org/>). A hierarchical annotation method was adopted to reduce human error. The annotation task consisted of two steps. In step1, a job-distribution system randomly assigned the task to one radiologist on the annotation board, that consisted of 10 radiologists with 2 or more years’ experience in cardiovascular imaging. The radiologist firstly annotated the heart region on the chest CT images and then annotated the calcified lesions on different coronary artery branches with different values (colors) at pixel-wise level, that was, lesions on the LAD, LCX, RCA, and LM branches were marked as 1, 2, 3, and 4, respectively. In step2, two radiologists with more than ten years’ experience in cardiovascular imaging jointly reviewed and revised the annotations in step1. Finally, all eligible scans were randomly divided into the training (70%), tuning (20%), and test (10%) datasets.

The proposed deep learning algorithm consisted of two steps, as shown in supplementary Figure 1. In step1, a modified 3D U-Net deep learning model was developed to accomplish the cardiac segmentation in order to reduce the computational cost and the false positive rate. The original 3D U-net architecture has four layers for encoder and decoder respectively, ^1^ to improve the architecture of 3D U-net, a Bottle-Neck design with 11, 33 and 11 convolution was added between each two layers. As we mainly focus on the position of the cardiac, this step required lower segmentation accuracy. After the cardiac was segmented by the model, the image within the cardiac area would be cut out and used as the input data for the next step. In step2, another 3D U-Net deep learning model was trained to segment coronary artery calcified lesions and classify them according to branches. In this step, the output target area not only contains information about whether the pixel is a calcified area, but also information about which branch of the coronary artery the pixel belongs to (the corresponding relationship of the gray value of the segmented image is LAD = 1, LCX = 2, RCA = 3, LM = 4). The structure of the deep learning model was consistent with the network structure in the previous step.


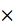

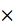

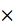

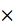

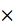

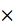


After obtaining the calcification segmentation results, the coronary artery calcium scores including the volume, mass, and Agatston score were calculated according to the previous studies.^2-6^

The performance of the proposed model was evaluated using dice similarity score (DSC), which represents the overlap ratio between the ground truth and segmentation results. In the test dataset (n=142), the final algorithm achieved a DSC of 0.9383 for the cardiac segmentation and a DSC of 0.8465 for the calcification segmentation.

**References** (Suppl.)

1. Çiçek Ö., Abdulkadir A., Lienkamp S.S., Brox T., Ronneberger O. (2016) 3D U-Net: Learning Dense Volumetric Segmentation from Sparse Annotation. In: Ourselin S., Joskowicz L., Sabuncu M., Unal G., Wells W. (eds) Medical Image Computing and Computer-Assisted Intervention – MICCAI 2016. MICCAI 2016. Lecture Notes in Computer Science, vol 9901. Springer, Cham.
2. Callister TQ, Cooil B, Raya SP, Lippolis NJ, Russo DJ, Raggi P. Coronary artery disease: improved reproducibility of calcium scoring with an electron-beam CT volumetric method. Radiology. 1998; 208:807–14.
3. Hong C, Bae KT, Pilgram TK. Coronary artery calcium: accuracy and reproducibility of measurements with multi-detector row CT--assessment of effects of different thresholds and quantification methods. Radiology. 2003; 227:795–801.
4. Hoffmann U, Siebert U, Bull-Stewart A, Achenbach S, Ferencik M, Moselewski F, et al. Evidence for lower variability of coronary artery calcium mineral mass measurements by multi-detector computed tomography in a community-based cohort--consequences for progression studies. Eur J Radiol. 2006;57:396–402.
5. Agatston AS, Janowitz WR, Hildner FJ, Zusmer NR, Viamonte MJ, Detrano R. Quantification of coronary artery calcium using ultrafast computed tomography. J Am Coll Cardiol. 1990;15: 827–32.
6. Sudre CH, Li W, Vercauteren T, Ourselin S, Jorge Cardoso M. Generalised Dice Overlap as a Deep Learning Loss Function for Highly Unbalanced Segmentations. In: Cardoso MJ, Arbel T, Carneiro G, Syeda-Mahmood T, Tavares JMRS, Moradi M, et al., editors. Deep Learning in Medical Image Analysis and Multimodal Learning for Clinical Decision Support. Cham: Springer International Publishing; 2017. p. 240–8.


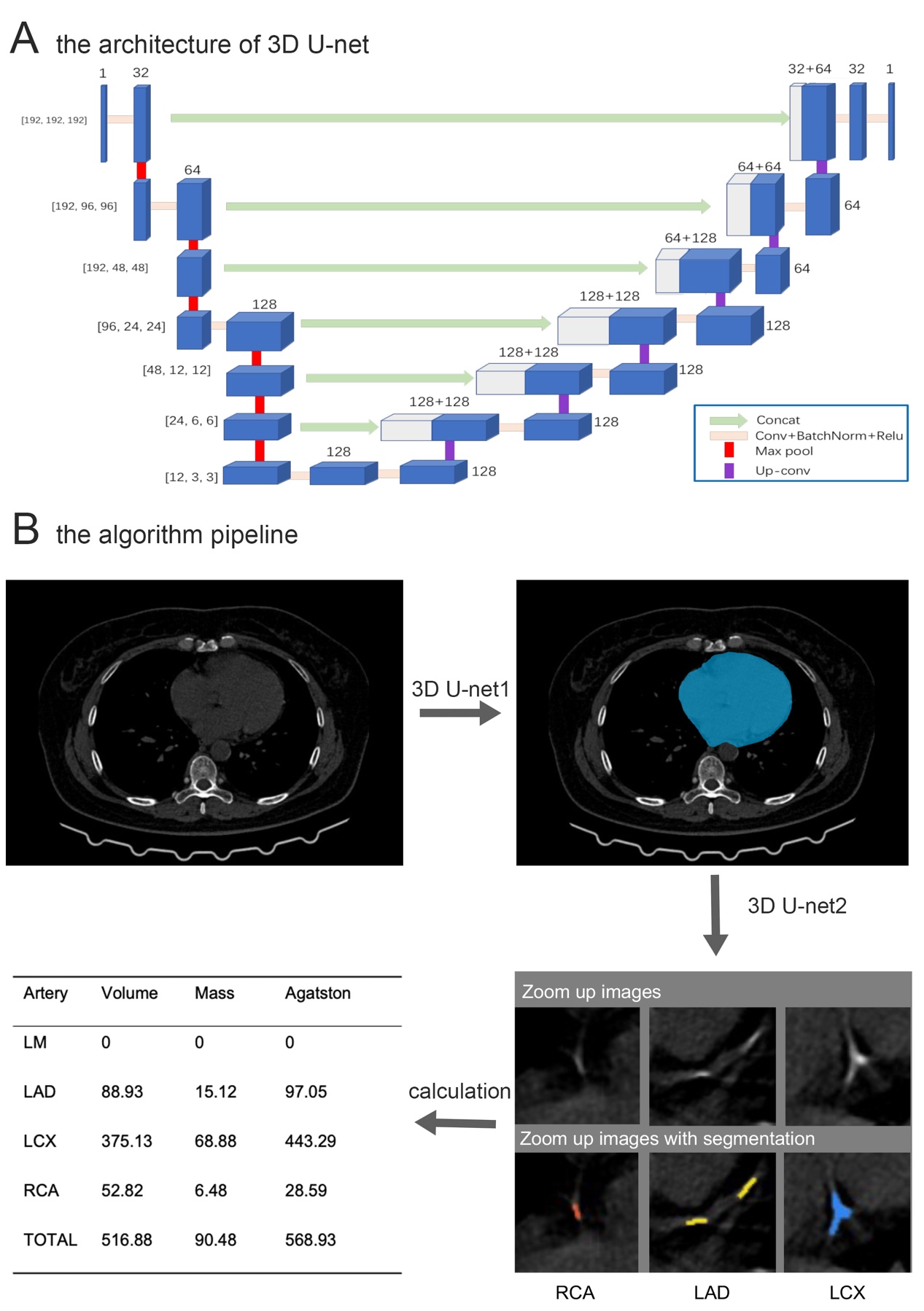


Figure 1. A. Schematic diagram to show architecture of 3D U-net used in this study. B. An example to show the pipeline of the deep learning-based algorithm to calculate CAC score.
