# Supplementary material for: Automated total and vessel-specific coronary artery calcium (CAC) quantification on chest CT: direct comparison with CAC scoring on non-contrast cardiac CT
Source: BMC Med Imaging. 2022 Oct 14;22:177. doi: 10.1186/s12880-022-00907-1 (PMC9563469; doi:10.1186/s12880-022-00907-1)
Supplement: Supplementary file 2 — Additional file 2: Manual measurement of Coronary artery calcium (CAC) distribution on chest CT. [file 12880_2022_907_MOESM2_ESM.docx]

**Additional File 2: Manual measurement of Coronary artery calcium (CAC) distribution on chest CT**

**Methods**

Visualization of CAC on non-gated chest CT was performed by a radiologist resident (W.S, >5 years of experience in cardiothoracic imaging) supervised by a senior radiologist (H.SH) with 20 years of experience in cardiothoracic imaging. The two radiologists were both blinded to the results of the ECG-gated scoring.

CAC lesions on chest CT were manually annotated to the left main trunk (LM), left anterior descending artery (LAD), circumflex (CX), and right coronary artery (RCA) non-gated chest CT using the semiautomatic software (CaScoring, Syngo. Via VB20; Siemens Healthineers, Erlangen, Germany) by two radiologists independently. The number of involved vessels (N1 to N4) was then determined. The discrepancies were resolved through discussion.

The sensitivity, specificity, false-negative rate, and false-positive rate of vessel-specific CAC detection were calculated using visualization on dedicated ECG-gated cardiac CT as a reference. The reliability of non-gated chest CT for manual identification of the number of involved vessels was determined by Cohen’s linearly weighted kappa, and agreement of classification was calculated.

**Results**

The per-vessel sensitivity, specificity, false-negative rate, and false-positive rate of non-gated CAC scoring were shown in Supplementary Table 1.

**Table 1.** Manual detection of individual coronary arteries with CAC.

ECG: electrocardiograph; LM: left main trunk; LAD: left anterior descending artery; LCX: circumflex; RCA: right coronary artery

CAC-: CAC absent, CAC+: CAC present.

FN: false-negative rate; FP: false-positive rate

The reliability of manual assessment of CAC regional distribution was strong on non-gated chest CT with a linearly weighted kappa of 0.82 (95%CI 0.78–0.86) and an assignment agreement of 79.0%, using standard CAC scoring on cardiac CT as a reference standard. (Supplementary Table 2)

**Table 2.** The number of vessels on chest CT versus measurements on cardiac CT

ECG: electrocardiograph

N1=number of 1-vessel CAC; N2=number of 2-vessel CAC; N3=number of 3-vessel CAC; N4=number of 4-vessel CAC; N0=no CAC involved

The further analyses by standard CAC category on cardiac CT were shown in Figure 1. The overall accuracy of the assignment was 52.4%, 65.1%, and 76.9% on non-gated chest CT for patients with standard CAC scores of 1-99 (A1), 100-299 (A2), and ≥300 (A3) respectively.


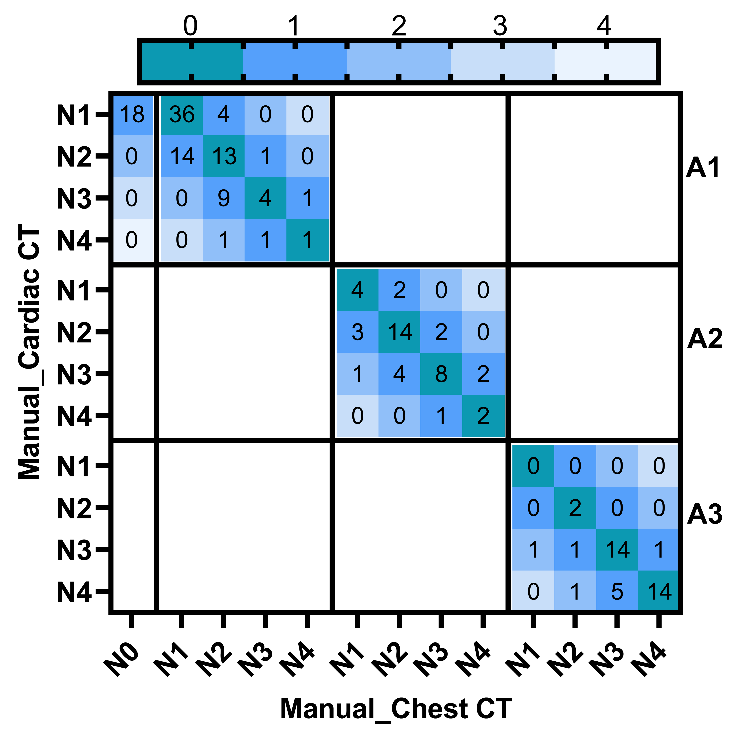


**Figure 1.** Comparison of the number of vessels between non-gated chest CT and ECG-gated cardiac CT.

The color bar on the top indicates the category shifts of the number of vessels (N1–N4) manually determined on chest CT compared to measures on cardiac CT. (0= correctly identification, 1=1vessel not identified, 2=2 vessels not identified, 3=3vessels not identified and 4=4 vessels are not identified).

The patients with positive CAC are classified into 3 groups according to standard CAC scores on ECG-gated cardiac CT (A1:1-99, A2:100-299, A3:≥300).Note that among patients of category A1, eighteen are falsely identified as N0 on chest CT.
